# Cardiovascular risk assessment - From individual risk prediction to estimation of global risk and change in risk in the population

**DOI:** 10.1186/1741-7015-8-29

**Published:** 2010-05-25

**Authors:** John A Batsis, Francisco Lopez-Jimenez

**Affiliations:** 1 Dartmouth Medical School, 1 Rope Ferry Road, Hanover, NH 03755, USA; 2 Division of Cardiovascular Diseases, Department of Medicine, Mayo Clinic College of Medicine, 200 First Street SW, Rochester, MN 55905, USA; 3 Section of General Internal Medicine, Department of Medicine, Dartmouth-Hitchcock Medical Center, 1 Medical Center Drive, Lebanon, NH 03756, USA

## Abstract

**Background:**

Cardiovascular disease is the most common cause of death and risk prediction formulae such as the Framingham Risk Score have been developed to easily identify patients at high risk that may require therapeutic interventions.

**Discussion:**

Using cardiovascular risk formulae at a population level to estimate and compare average cardiovascular risk among groups has been recently proposed as a way to facilitate surveillance of net cardiovascular risk and target public health interventions. Risk prediction formulas may help to compare interventions that cause effects of different magnitudes and directions in several cardiovascular risk factors, because these formulas assess the net change in risk using easily obtainable clinical variables. Because of conflicting data estimates of the incidence and prevalence of cardiovascular disease, risk prediction formulae may be a useful tool to estimate such risk at a population level.

**Summary:**

Although risk prediction formulae were intended on guiding clinicians to individualized therapy, they also can be used to ascertain trends at a population-level, particularly in situations where changes in different cardiovascular risk factors over time have different magnitudes and directions. The efficacy of interventions that are proposed to reduce cardiovascular risk impacting more than one risk factor can be well assessed by these means.

## Background

### Prediction of CV risk

As cardiovascular (CV) disease corresponds to the most common cause of death in the United States with estimates exceeding one million deaths annually [1], estimates of individual and population-based CV risk are of paramount importance. CV risk prediction formulae and tables are decision tools that allow the identification of patients at high risk of CV disease. These tools allow early interventions by providers to recommend lifestyle modification or drugs to control modifiable CV risk factors, including hypertension, diabetes, smoking, dyslipidemia and obesity.

Several CV risk prediction formulae are used in clinical practice worldwide. In the United States, the modified Framingham Risk Score (FRS) is the most commonly used tool [2], and has been adapted for use in diverse populations in other parts of the world. Other tools include the Prospective Cardiovascular Munster Heart Study (PROCAM) [3], the Systematic Coronary Risk Evaluation system (SCORE) [4], United Kingdom Prospective Diabetes Study (UKPDS) [5] tool for diabetics, the Reynolds Risk Score [6,7] and more recently, one which includes obesity as a variable (NHANES) [8].

The variables included in the FRS include age, sex, smoking status, diabetes status, cholesterol, and blood pressure values. These variables are routinely available in patients receiving medical care, particularly in a primary care setting, as routine screening for hypertension, smoking status, dyslipidemia, and fasting hyperglycemia are part of normative preventative health measures [9]. With such information clinicians could either use gender-specific risk score tables in assigning points that can translate into a given 10-year CV risk, or use electronic or web-based risk calculators http://www.framinghamheartstudy.org/risk/coronary.html to calculate such risks. The purpose of risk stratification is to identify and treat patients that may be at higher long-term CV risk in a simple and cost-efficient manner. This risk tool has an acceptable area under the receiver operating curve of roughly 75% [10]. Most of these tools, particularly the FRS have been validated in many different populations and ethnic groups and recalibrated appropriately [11-14], making it a well-known risk index that allows comparison of risks across different population groups. However, there are distinct ethnic populations, particularly in those with higher prevalences of metabolic syndrome where recalibration is often challenging and its applicability may be limited [11,13-15].

The advent of other markers of CV disease, including high sensitivity C-reactive protein (HS-CRP), homocysteine, lipoprotein (a), or coronary calcification scores [16-18], have been shown to predict incident coronary disease but they add little prognostic value to standard risk formulae and their incorporation into present clinical practice has been challenging. High sensitivity CRP is associated with an increased risk of future cardiovascular disease, diabetes and even hypertension [16,19,20]. Yet studies have shown a minimal incremental value of adding new biomarkers to existing prediction models in a recent study by the Framingham group [21]. Interestingly, there is emerging evidence in potentially using CRP, particularly using the Reynolds Risk Score, to re-classify intermediate risk subjects into a high risk category for potential interventions [6,7,22]. In addition, detection of subclinical coronary artery disease with screening tests like CT to measure coronary calcium has been a topic of a recent debate. The AHA Consensus document which outlined the likely benefit of using this modality to risk-stratify intermediate risk patients to either high or low categories depending on the score [23]. This group did not recommend its use in either low or high-risk patients. The utility of biomarkers for the detection of subclinical coronary disease for risk stratification may be limited in individuals believed to be at high CV risk, who for some reason have an FRS that is not too high, but these biomarkers are possibly useful in those with intermediate risk.

## Discussion

### Limitations of studies assessing the prevalence of coronary artery disease

The assessment of disease burden in the population is of critical importance for public health officials and health care policy makers. In the case of CV disease burden, there are some challenges to estimate changes in incidence and prevalence of CV disease. Although some epidemiologic studies have shown a downtrend in CV mortality in the USA [24-26], the trend in incident CV disease has demonstrated conflicting results [27]. Some of the problems are the change in diagnostic criteria for myocardial infarction, changes in screening patterns and improved diagnostic modalities to detect coronary and vascular disease in the subclinical phase, factors that will directly affect the likelihood to diagnose a person with CV disease.

Thus, estimates of change in CV risk may be meaningful and valid alternatives to assess trends in CV burden in the population. If CV risk prediction formulae have been shown to be accurate and useful in estimating risk at the individual level, they may also provide estimates of CV risk at the population level. The total CV risk in a population can be obtained by calculating the average CV risk using individual-based information on CV risk factors from nationally representative data like the National Health and Nutrition surveys (NHANES) and weighting it for standardized demographics [28].

The value of CV risk prediction formulae to estimate risk at the population level is also justified because CV risk factors have been constantly changing in different magnitude and direction over the past 30 years. For example, mean cholesterol values showed a downward trend soon after statins became widely available, but then flattened years later [29]. A similar trend was observed for cigarette smoking [29,30], while other factors like obesity [31,32] and diabetes mellitus [33-35] have become more prevalent. Furthermore, changes have not been uniform for both sexes and across different age strata. Because these major CV risk factors provide different strengths of risk for incident myocardial infarction [36], the only way to know the net trend in risk for incident CV disease in a given country may be by using risk prediction formulae.

A recent analysis examining changes in the predicted 10-year CV risk in the US, demonstrated that the estimated net risk for CV disease in the US population decreased between 1976 to 1980 and 1988 to 1994, but has changed minimally from 1988 to 1994 and from 1999 to 2004, particularly in women and middle-aged people [28]. These data have enormous public health implications, and suggest that the gain in primary prevention of CV disease that occurred from 1976 to 1980 and 1990 to 1994 has levelled off during the last time period, despite the discovery and implementation of effective treatment modalities in managing dyslipidemia and hypertension [37-39], national anti-smoking campaigns [40] and efforts related to primordial prevention. This study confirmed the utility of using simple risk-prediction tables or equations to project future CV risk and assess the trajectory of given trends. In addition, the same principle of usability of risk scores to assess population trends in predicted cardiovascular health may be also applied to newer scores attempting to predict lifetime CV risks [41]. In this study, aimed at predicting lifetime CV risk, the majority of participants (56%) were classified as having a low short-term risk using the FRS. Indeed, using newer risk prediction rules that go beyond the typical 10-year horizon may have greater meaning for public health and public policy. Policy makers should be actively analyzing existing epidemiologic datasets in order to assess ongoing trends in net CV risk and to determine the areas with the highest yield to reduce CV risk in the population and plan for public health interventions.

### Estimation of net change in CV risk for interventions that affect risk factors in different ways

Although pharmacologic interventions for either hypertension or dyslipidemia can induce a significant reduction in CV risk, lifestyle interventions that affect several risk factors are also a critical step in managing CV risk. Different lifestyle interventions lead to different levels of change in lipids, blood pressure, body weight and blood glucose, and therefore the net change in cardiovascular risk after implementing these interventions is rarely known. The comparison of different strategies in reducing CV risk is also problematic when they affect different CV risk factors in different ways. For example, very low fat diets can induce a reduction in body weight, blood pressure, and total and LDL cholesterol, but may also cause a reduction in HDL cholesterol. Mediterranean diets may improve weight, blood pressure and lipids, but cause HDL cholesterol to rise, although their effect on LDL may be more modest [42]. Diets like the dietary approaches to stop hypertension (DASH) also affect lipids, but their major benefit comes from blood pressure reduction, with limited effect on fasting glucose [43]. Thus, to ascertain which diet has a more favorable effect on CV events it is necessary to estimate and compare the net change in CV risk. Surprisingly, there have been limited studies examining the impact of either dietary modification or physical activity on 10-year predicted CV risk. Multiple studies have examined the impact of CV risk factor reduction following pharmacologic interventions, but very few have ascertained the net change in CV risk. Specifically, changes in Framingham risk with lifestyle intervention have not been extensively studied. One study demonstrated modest changes in FRS for primary prevention of CV disease using a health report card with counselling on cardiovascular risk factors [44]. The largest prospective trial to date recently evaluated a multi-component lifestyle intervention demonstrating a relative risk of 0.88 (0.83 to 0.94; *P *< 0.001) in patients undergoing an established lifestyle recommendation for blood pressure control (reduction in salt, weight loss, and physical activity) with a DASH diet, and 0.86 (0.81 to 0.91; *P *< 0.001) in the established lifestyle alone using the FRS [45].

### Estimation of events prevented with specific interventions

Cardiovascular (CV) risk prediction formulae provide an estimated value that corresponds to the likelihood of developing CV events over a period of time. Thus, it could be argued that interventions that reduce the estimated risk would also result in a reduction in CV events. Two studies were published examining change in CV risk using both the FRS but also a risk score derived from the National Health and Nutrition Examination Surveys [8,46]. Using the FRS, the relative risk reduction for a cardiovascular event in patients managed surgically for obesity was 50%, while all-cause mortality was reduced by 44.2% using the NHANES risk score. The 10-year risk did not change from 30% at baseline to 30% at follow-up in non-operative controls [8]. Such an intervention represents an estimate of 4 overall deaths and 16 cardiovascular events prevented by bariatric surgery per 100 patients compared with the non-operative group.

Other studies, using bariatric surgery cohorts, have used the FRS to estimate the CV risk reduction, and have demonstrated a relative risk reduction of 33% with an absolute reduction in FRS score from 6% to 4% (*P *< 0.001) [47]. Recently, two studies have examined the impact of bariatric surgery on long-term patient outcomes whose results parallel the outcomes predicted in the above studies. The Swedish Obesity study, whose study population consists primarily of vertical banded gastroplasty patients, had prospective patient data and the adjusted HR for death in the surgical group was 0.71, suggesting a 29% less risk of death at 10-years in the surgical group [48]. In the study by Adams *et al*. [49], the adjusted long-term mortality was 40% lower in the surgical group with 7.1 years of follow-up. These results suggest that prediction tools like the FRS can potentially be practically used to estimate actual number of events in patients at higher cardiometabolic risk. We do caution, though, that further studies are needed to validate the use of such formulae in such select populations which may not be representative of the cohorts that the original formulae were developed from, to additionally incorporate disease-specific or procedure-specific complications in these assessments.

### Shortcomings of current risk prediction tools

The FRS relies on traditional risk factors including hypertension, diabetes, smoking status, and dyslipidemia. Other risk factors like family history of premature CV disease, obesity, high sensitivity CRP, inflammatory cytokines and lifestyle are not incorporated in present risk formulae. By omitting these risk factors, the current risk prediction formulas may underestimate CV risk in some individuals, particularly those at intermediate CV risk [6,7,22].

Accurate risk equations are often translated into risk tables to facilitate clinical decision making. Tables are used clinically to differentiate between high and low risk patients, allowing the implementation of preventative strategies. Thresholds are often used to categorize continuous variables as normal or abnormal or using different level proportional risk using a points system. By doing so, some prognostic information in extreme values is disregarded despite the linear or near linear association between CV risk factors and CV events. Variables whose incremental risk is proportional or exponential to its level, for instance, with hypertension or elevated LDL, the dichotomization of variables according to a given threshold leads to similar risks being applied to values that barely exceed the threshold with those that are far beyond the threshold. Conversely, values that are slightly below the threshold are often treated as normal, which leads to inaccuracies.

Sometimes, therapeutic interventions may have unintended consequences on other diseases, such as the increased mortality rates observed in diabetes with intensive insulin regimens [50]. The intent of such interventions is to improve cardiovascular risk factors which may not translate in a reduction in clinical events. In addition, other interventions, such as sodium restriction have larger effects on CV disease than its impact on blood pressure alone and modest effects yield greater than expected changes, which extend beyond such clinical markers [51]. This may limit the use of such prediction rules in forecasting trends in CV events using information related to changes in CV risk factors. Future scores should acknowledge and address these limitations.

However, further research is needed to be able to apply these risk prediction tools, intended for individualized patients, to translate into larger population-based health interventions. As the degree of cardiovascular and overall morbidity and mortality changes, the necessity of recalibrating existing risk equations is needed. For instance, the Framingham equation was based on a population of patients decades ago whose characteristics are much different than those existing nowadays [2]. In addition, with the development of new biomarkers and enhanced understanding of the pathophysiologic of atherosclerosis, future formulae will likely incorporate measures of factors representing different mechanistic pathways beyond the current approach that addresses traditional risk factors only. Hence, clinicians and public health officials need to be aware of the population-level estimates of disease burden to assess absolute risk. Furthermore, calibration needs to occur to allow application of risk algorithms to other population and/or ethnic groups. More importantly, risk prediction equations target preventative care to those asymptomatic patients at intermediate or high risk, but still roughly a third of all CV events occur in subjects labeled as low risk by common equations.

## Summary

Risk prediction formulas were originally created for proper CV risk stratification at the individual level. They are recommended to guide medication therapy and identify people in whom interventions may be more cost-effective. The use of such risk prediction formulas in assessing risk trends at a population level may also be used to predict changes in net CV risk, potentially predicting changes in the incidence of CV disease and therefore may be used to preliminarily assess the trend in CV disease risk and burden in a particular population. These formulas can also be used to assess net benefit in CV risk reduction for interventions that can affect several CV risk factors such as diets with different macronutrient compositions that affect different CV risk factors, in different directions and magnitudes. Population-based estimates, though, can be guided by such formulae, understanding the caveat that not only calibration is needed when predicting risk in different populations, but also to consider the shortcomings due to imperfect prediction of clinical outcomes.

## Abbreviations

CV: cardiovascular; FRS: Framingham risk score; HDL: high density lipoprotein; HR: hazard ratio; HS-CRP: high sensitivity C-reactive protein; LDL: low density lipoprotein; NHANES: National Health and Nutrition Examination Survey; PROCAM: Prospective Cardiovascular Munster Heart Study; SCORE: Systematic Coronary Risk Evaluation system; UKPDS: United Kingdom Prospective Diabetes Study.

## Competing interests

The authors declare that they have no competing interests.

## Authors' contributions

The authors contributed equally to the above manuscript in the conception, design, drafting and critically revising the manuscript, in addition to approving the final version.

## Pre-publication history

The pre-publication history for this paper can be accessed here:

http://www.biomedcentral.com/1741-7015/8/29/prepub
